# The Relationship between Fine Particle Matter (PM2.5) Exposure and Upper Respiratory Tract Diseases

**DOI:** 10.3390/jpm14010098

**Published:** 2024-01-16

**Authors:** Łukasz Zaręba, Katarzyna Piszczatowska, Karolina Dżaman, Karolina Soroczynska, Parham Motamedi, Mirosław J. Szczepański, Nils Ludwig

**Affiliations:** 1 Department of Biochemistry, Medical University of Warsaw, 02-097 Warsaw, Poland; lukasz.zareba@wum.edu.pl (Ł.Z.); katarzyna.piszczatowska@wum.edu.pl (K.P.); karolina.soroczynska@wum.edu.pl (K.S.); s081981@student.wum.edu.pl (P.M.); 2 Department of Otolaryngology, The Medical Centre of Postgraduate Education, 03-242 Warsaw, Poland; kfrydel@poczta.onet.pl; 3 Department of Oral and Maxillofacial Surgery, University Hospital Regensburg, 93053 Regensburg, Germany

**Keywords:** PM2.5, allergic rhinitis, rhinosinusitis, airborne pollution, upper respiratory tract

## Abstract

PM2.5 is one of the most harmful components of airborne pollution and includes particles with diameters of less than 2.5 μm. Almost 90% of the world’s population lives in areas with poor air quality exceeding the norms established by the WHO. PM2.5 exposure affects various organs and systems of the human body including the upper respiratory tract which is one of the most prone to its adverse effects. PM2.5 can disrupt nasal epithelial cell metabolism, decrease the integrity of the epithelial barrier, affect mucociliary clearance, and alter the inflammatory process in the nasal mucosa. Those effects may increase the chance of developing upper respiratory tract diseases in areas with high PM2.5 pollution. PM2.5’s contribution to allergic rhinitis (AR) and rhinosinusitis was recently thoroughly investigated. Numerous studies demonstrated various mechanisms that occur when subjects with AR or rhinosinusitis are exposed to PM2.5. Various immunological changes and alterations in the nasal and sinonasal epithelia were reported. These changes may contribute to the observations that exposure to higher PM2.5 concentrations may increase AR and rhinosinusitis symptoms in patients and the number of clinical visits. Thus, studying novel strategies against PM2.5 has recently become the focus of researchers’ attention. In this review, we summarize the current knowledge on the effects of PM2.5 on healthy upper respiratory tract mucosa and PM2.5’s contribution to AR and rhinosinusitis. Finally, we summarize the current advances in developing strategies against PM2.5 particles’ effects on the upper respiratory tract.

## 1. Introduction

According to the WHO, globally, 4.2 million people die prematurely every year from outdoor ambient air pollution. Almost 90% of the world population permanently lives in areas with poor air quality that does not meet the standards established by the WHO [1]. The components of ambient air pollution that are considered dangerous to health include particulate matter (PM), ozone, nitrogen dioxide (NO_2_), sulfur dioxide (SO_2_), carbon monoxide (CO), various organic compounds, e.g., benzene, and more [2,3]. PM particles are divided based on their size into PM10 (particle diameters less than 10 μm), PM2.5 (particle diameters less than 2.5 μm), and PM0.1 (particle diameters less than 0.1 μm) [3]. The size of the PM is correlated with the level of upper and lower respiratory tract that they get trapped in. PM10 tends to accumulate in the upper respiratory tract while PM2.5 and PM0.1 more often reach the structures of lower respiratory tract resulting in respiratory and systemic diseases [4].

It was shown that the location of PM collection highly affects its chemical composition. For instance, the percentage of the traffic exhaust in the PM2.5 composition differs based on the location such as traffic sites, urban background sites, and rural sites. Other important PM2.5 contributors include non-traffic exhaust, fuel oil combustion, biomass burning, soil dust, sea salt, and secondary aerosols [5]. Additionally, the composition of PM2.5 varies even among comparable types of areas. For example, Salameh et al. demonstrated that PM2.5 collected in five different Mediterranean European cities contained distinct percentages of the same components [6]. Moreover, the median PM2.5 concentrations vary depending on the geographical location and the time of year. Some cities in Asia struggle with higher medial PM2.5 concentrations than those located in other parts of the globe [7]. The PM2.5 concentrations in urban areas tend to be higher in winter than in other seasons of the year [8]. These fluctuations of the concentration in urban areas could be attributed to a variety of factors, including traffic, synoptic conditions, or increased heating during winter [9]. Interestingly, the COVID-19 pandemic has resulted in a decrease in PM2.5 concentrations in city environments, which might be explained by the lockdowns and social restrictions [10].

PM2.5 exposure has been related to a wide range of disorders impacting numerous systems such as the cardiovascular, respiratory, immune, and even reproductive system, which may result in an increased risk of infertility [11,12,13,14]. Many cardiovascular diseases, such as atherosclerosis, ischemic heart disease, and stroke, have been associated with PM2.5 exposure [15,16]. It has been also suggested that PM2.5 may cause immunological impairments leading to a weakened host defense [14]. Additionally, there is a wide group of respiratory pathologies that are induced or exacerbated by PM2.5 exposure. Many of them are attributed to the lower respiratory tract, such as asthma, chronic obstructive pulmonary disease (COPD), infections, or lung cancer [17,18,19,20].

The effect of PM2.5 on the upper respiratory tract has recently sparked a lot of attention, and the number of papers on the subject is continually expanding. The evidence suggests that PM2.5 exposure may contribute to the induction or exacerbation of allergic rhinitis (AR) and rhinosinusitis [21,22]. The main tissue of the upper respiratory tract affected by PM2.5 particles is the epithelia of the nasal and paranasal sinuses [23,24]. Many studies have recently explored the effect of PM2.5 particles on nasal and sinonasal epithelial cells, resulting in the discovery of a variety of mechanisms underlying the detrimental PM2.5 effects [23,24,25,26]. The local pathological activity of particles on the epithelium can be divided into four categories: impairment of cell metabolism, adverse impact on epithelial barrier integrity, dysfunction of mucociliary clearance (MCC), and provoking of an increased local inflammatory response [23,25,26]. Furthermore, several studies have pointed out that PM2.5 exposure affects the composition of the microbiota in the nasal cavity and pharynx which could subsequently affect epithelial function [27,28]. These pathological mechanisms may be responsible for the outcomes that were seen in patients with AR and rhinosinusitis who exhibited an exacerbation of their disease after exposure to higher PM2.5 concentrations [29,30]. In this review, we focus on the effect of PM2.5 particles on the upper respiratory tract by pointing out the most important pathological mechanisms triggered by PM2.5 and discussing its impact on upper respiratory tract diseases. Furthermore, we discuss potential future directions in fighting the adverse impact of PM2.5 on the upper respiratory tract. 

## 2. PM2.5 Effects on Healthy Nasal and Sinonasal Epithelia

### 2.1. Disruption of Epithelial Cell Metabolism

Nasal and sinonasal epithelial cells form a barrier that serves as an essential defensive mechanism against particles of air pollution. The efficiency of this barrier is determined by a variety of factors, including the appropriate functioning of the epithelial cells found in the nose and paranasal sinuses. Some of the most important factors that contributes to cell integrity and homeostasis are energy metabolism and proper reactive oxygen species (ROS) clearance [31,32]. The affected mitochondrial function and imbalance in ROS production may lead to the disruption of tight junction proteins and contribute to chronic rhinosinusitis [33,34].

Oxidative stress in nasal epithelial cells was classified as an adverse effect of PM2.5 exposure [25,34]. The increased level of ROS, which causes oxidative stress, may occur due to several mechanisms. One of them is the alteration of antioxidant enzyme activity. Several studies confirmed that PM2.5 exposure reduced superoxide dismutase (SOD) activity and increased the level of the oxidative stress marker malondialdehyde (MDA) in the nasal mucosa [25,35,36]. Moreover, Hong et al. demonstrated that RPMI 2650 cells exposed to PM2.5 exhibited decreased activity of other antioxidant enzymes such as catalase (CAT) and glutathione peroxidase (GSH-Px). Interestingly, the levels of nuclear factor erythroid 2-related factor 2 (Nrf2), a key regulator of cellular oxidant resistance, were increased in the nucleus and decreased in the cytoplasm after PM2.5 treatment, suggesting that the nuclear translocation of Nrf2 was inhibited [35,37]. Analogous to these findings, Hong et al. demonstrated that GSH-Px was downregulated in the nasal mucosa of rats exposed to PM2.5 and Gu et al. demonstrated that PM2.5 exposure decreased GSH and increased MDA levels in human nasal epithelial cells [36,38].

Mitochondrial damage often occurs together with induced oxidative stress [39]. The studies of Guo et al. revealed that the structure of mitochondria in rats’ nasal epithelial cells was altered after PM2.5 exposure, resulting in mitochondrial swelling, mitochondrial cristae disorder, swelling membrane breach, or vacuolization [25]. Furthermore, the levels of mitochondrial mRNAs and their proteins such as mitochondrial dynamin-like GTPase (OPA1) and mitofusin 1 (MFN1) were decreased after treatment with PM2.5 in small and moderate concentrations and increases after treatment with high PM2.5 concentrations. Dynamin-related protein 1 (Drp1) and mitochondrial fission 1 protein (Fis1) mRNAs and their protein levels were increased after exposure to high PM2.5 concentrations and ultimately, these alterations in mRNA and protein levels were all related to the mitochondrial dysfunction [25]. Jia et al. also observed signs of mitochondrial damage such as clarification of the mitochondrial matrix and destruction of the integrity of the mitochondrial membrane and cristae in epithelial cells after PM2.5 administration in vitro [40].

Finally, studies have shown that the morphological and functional changes in epithelial cells after PM2.5 exposure led to decreased cell viability [35,41]. This effect could be directed by multiple changes in mitochondria metabolism and increased oxidative stress. Interestingly, PM2.5 reduced the viability of nasal epithelial cells by affecting iron metabolism, lipid peroxidation, and autophagy. Gu et al. showed that PM2.5 increased lipid peroxidation and the iron content in nasal epithelial cells. It was found that PM2.5 exposure induced phosphorylation of AMPKα and reduced expression of ferroptosis-related proteins such as GPx4, xCT, FTH1, and FTL. These changes led to autophagy and ferroptosis induction in nasal epithelial cells [38].

In summary, PM2.5 particles affect mitochondrial function and disrupt ROS clearance which is a toxic effect that may provoke stress and other adverse effects such as damage to intercellular connections, dysfunction of mucus production and cilia movements, or exacerbated local inflammatory process [33,42].

### 2.2. Dysfunction of Intercellular Connections

The next important factor that contributes to nasal and sinonasal epithelial barrier efficiency is the proper structure of cell–cell connections such as tight junctions (TJs) and adherens junctions (AJs) [43]. TJs are located in apical parts and consist of protein families including claudins, occludins, catenins, and zonula occludens (ZO) [44,45]. AJs are present below TJs in the epithelial barrier and are comprised of cadherin and nectin family proteins [46].

Several studies demonstrated that PM2.5 particles affected the structure of TJs and AJs [23,24]. Xian et al. demonstrated that in vitro PM2.5 exposure reduced occludin, claudin-1, and ZO-1 expression levels in nasal epithelial cells. Moreover, the nasal mucosa obtained from the middle turbinate or the uncinate process of patients that underwent sinonasal surgery unconnected with inflammatory conditions revealed alterations in TJ-related proteins. The samples obtained from patients, who were operated on during winter, exhibited reduced immunoreactivity of occludin, claudin-1, and ZO-1 proteins compared to the patients operated on during summer. Moreover, the signal of TJ-related mRNAs including ZO-1, ZO-2, CLDN-1, CLDN-4, CLDN-7, and occludin was reduced in the winter samples [23]. During winter, the concentrations of PM2.5 in the air are much higher, which suggests that PM2.5 exposure may have played a role in the reported results of Xian et al. Another study conducted by Ramanthan et al. demonstrated that PM2.5 exposure decreased the expression of the TJ-related protein claudin-1 and the AJ-related E-cadherin in the nasal epithelial barrier of mice [24]. Moreover, Zhao et al. demonstrated that PM2.5 exposure reduced the barrier function of RPMI 2650 nasal epithelial cells. It was observed that PM2.5 reduced the levels of TJ-related ZO-1, occludin, and claudin-1 proteins in nasal epithelial cells [41].

These findings suggest that PM2.5 exposure affects the TJs and AJs between nasal and sinonasal epithelial cells, which may lead to the loss of integrity of the epithelial barrier in the nasal and paranasal sinuses.

### 2.3. Mucociliary Clearance Dysfunction

The nasal and sinonasal epithelium is the first layer of protection for the airways and is responsible for clearing airborne particles from inhaled air. This protective effect is provided by mucociliary clearance (MCC), a process in which, airborne particles are captured in the mucus layer and removed by cilia beating activity [47]. There are two main factors responsible for the success of MCC: the efficient production of mucus by goblet cells and submucosal glands, and an active ciliary beat of epithelial cells [47,48]. When ciliated epithelial cells or mucus production are dysfunctional, the effectiveness of the whole MCC mechanism is drastically decreased. Recently, a variety of studies have described an adverse effect of PM2.5 on nasal and sinonasal epithelial cells that may lead to MCC impairment [25,40].

Cilia disruption is one of the main adverse effects that occurs in epithelial cells exposed to PM2.5 particles. PM2.5 particles can change the structure and arrangement of nasal epithelial cilia, as demonstrated in the study conducted by Guo et al. [25]. Rats exposed to increasing concentrations of PM2.5 displayed a disarray of epithelial cilia in a dose-dependent manner. Furthermore, rats exposed to the highest concentrations of PM2.5 exhibited a total annihilation of cilia structures in their nasal epithelial cells [25]. Another study conducted by Jia et al. demonstrated that PM2.5-dependent cilia disruption occurred with functional impairments. Human nasal epithelial cells (HNEpCs) co-cultured with PM2.5 exhibited a higher ciliary beat frequency (CBF) after 12 h regardless of the PM2.5 concentration, whereas HNEpCs exposed to a higher dose of PM2.5 showed a decrease in CBF after 24 h [40]. These data suggest that PM2.5 exposure can provoke increased cilia activity, but longer exposure to the high PM2.5 concentration may induce cilia impairment.

**Figure 1:**
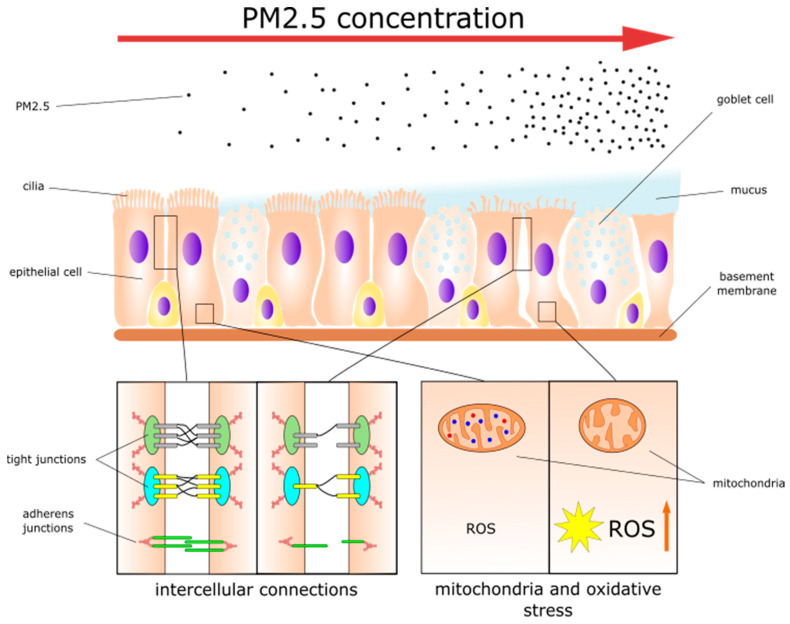
The effects of PM2.5 on nasal epithelial cells.

Mucus secretion is one of the two main factors involved in MCC success and goblet cells, as well as the submucosal glands, play a major role in mucus production [49]. The overall function of mucus is to catch airborne particles, irritants, and pathogens and deliver them to drainage sites via cilia motions [50,51]. Mucus mainly consists of water (95%) and other components such as mucin glycoproteins, lipids, other proteins, and salt [52]. Mucin glycoproteins are the most abundant proteins in the mucus and are responsible for forming a gel. Among them, two mucins are predominant: MUC5A and MUC5B [45,53]. Mucus also contains immunological components such as immunoglobulin A, lysozymes, and lactoferrin [51]. Any alterations in the mucus composition can be harmful to the local epithelium and ultimately to the entire organism. It was demonstrated that PM2.5 exposure may increase the production of acidic mucosubstances in healthy nasal epithelia [54]. Changes in the composition such as acidification of the mucus may cause impaired host defenses and increase the presence of airway bacteria [55]. Moreover, Gu et al. showed that PM2.5 caused increased mucus secretion in the nasal mucosa of mice [38]. The effects of PM2.5 on nasal epithelial cells are summarized in Figure 1.

### 2.4. Inflammatory Response in the Epithelium

The PM of airborne pollution irritates the cells of the nasal epithelium, which causes an activation of local inflammatory responses in the form of increased cytokine release and modulation of inflammatory cells [56]. Several studies revealed that this mechanism also occurs after PM2.5 exposure and inflammatory changes are one of the most common effects that occur in epithelial cells [23,24,25,35,57].

The local inflammation process triggered by PM2.5 leads to the release of many cytokines and interleukins involved in further modulation of inflammatory cells. The secretion of pro-inflammatory cytokines is one of the most basic protective mechanisms of the epithelial barrier defense [58]. Xian et al. showed this effect in cultures of primary human nasal epithelial cells that were exposed to PM2.5. Cells co-cultured with 100 μg/mL PM2.5 released significantly more inflammatory cytokines such as IL-8, TIMP-1, and TSLP [23]. An increase in IL-8 expression was also observed by Hong et al. who evaluated the effect of PM2.5 on the RPMI 2650 cell line [35]. Furthermore, they showed that in vitro PM2.5 exposure increased the release of proteins including GM-CSF, TNF-α, IL-13, eotaxin, and IL-6 [35]. Moreover, in a different study investigating the nasal lavage fluid (NLF) collected from PM2.5-exposed mice, an increased expression of the pro-inflammatory cytokines IL-1 beta, eosinophil-promoting IL-13, and eosinophil chemokine eotaxin-1 compared to the unexposed mice was noticed [24]. Guo et al. demonstrated similar results in NLF collected from healthy rats exposed to PM2.5, which contained higher levels of IL-4, IL-13, and TGF-β1 compared to the control NLF [25]. Also, Hong et al. showed that PM2.5 increased IL-1, IL-6, and TNF-α levels in the nasal mucosa of rats [36].

**Figure 2:**
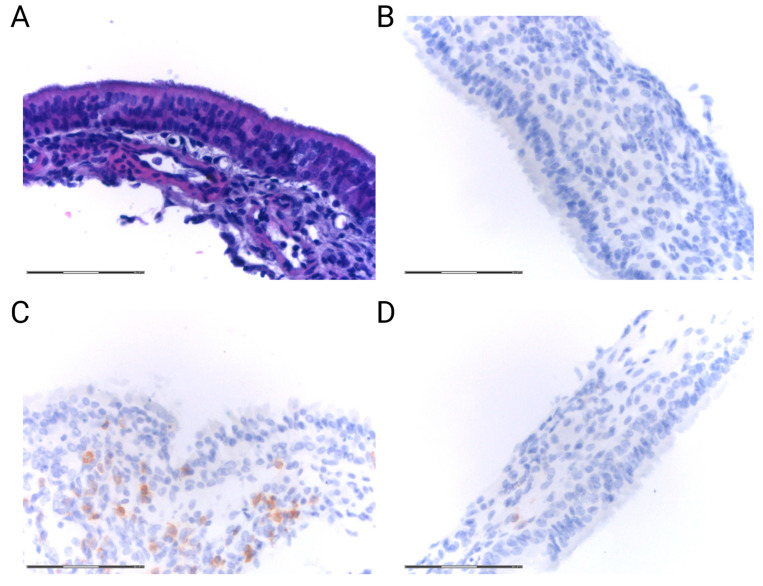
Representative H&E staining and immunohistochemistry staining for CD3 of mucosa harvested from C57BL/6 mice. Images were captured at 400× magnification. (**A**) H&E staining of mucosa from mice. (**B**) Negative control for CD3 staining of mice mucosa. (**C**) CD3 staining of mucosa from mice after inhalation of 3000 μg/m^3^ PM2.5. (**D**) CD3 staining of mucosa from mice that inhaled NaCl (unpublished observations) (scale bar = 75 μm).

Another crucial mechanism taking place during the local inflammatory response in the epithelium is inflammatory cell infiltration. Interestingly, exposure to high PM2.5 concentrations induced the infiltration of neutrophils and lymphocytes into the nasal mucosa of healthy rats [25]. This observation is in line with our preliminary data obtained from experiments with mice. C57BL/6 mice inhaled PM2.5 at three different concentrations, i.e., 25 μg/m^3^, 200 μg/m^3^, and 3000 μg/m^3^, for 30 days and 2 h per day and were compared to mice inhaling saline. The immunohistochemical analysis revealed an increased infiltration of CD3+ cells into the nasal mucosa of mice in response to PM2.5 exposure (Figure 2).

## 3. Contribution of PM2.5 to Upper Airway Disorders

### 3.1. Allergic Rhinitis

Allergic rhinitis (AR) is a prevalent condition and estimations suggest that it affects 20–30% of adults and up to 40% of children [59,60]. AR is the most frequent condition among the atopic disorders. Patients in most cases present nasal congestion, rhinorrhea, sneezing, and nasal itching, but many other related symptoms may appear as well [61]. The ARIA (Allergic Rhinitis and its Impact on Asthma) classification divides AR by severity, i.e., mild, moderate, and severe, and by occurrence type, such as intermittent or persistent [62].

AR is caused by sensitization which is the patient’s first contact with an allergen. During this process, epithelial cells absorb the allergen and release chemokines such as CCL20 and interleukins including IL-33, TSLP, and IL-25 that promote the recruitment of dendritic cells (DCs) [63]. After being presented with the allergen, DCs activate and migrate to the local lymph nodes where they stimulate CD4+ T cells to differentiate into allergen-specific Th2 cells. These cells produce IL-4, IL-5, and IL-13 which can stimulate B cells to differentiate into IgE-producing plasma cells [62,63,64,65]. After re-exposure to the allergen, activated memory B cells produce IgE which binds to the FcεRI receptor on mast cells and basophils leading to the early phase response [63].

IgE-dependent degranulation of basophils and mast cells is a key reaction in early phase response [63,66]. Released mediators such as leukotrienes and histamine lead to increased vascular permeability, rhinorrhea, itching, and increased mucus secretion [65]. The late phase response occurs 4–8 h after allergen administration and is characterized by inflammatory cell infiltration into the mucosa, including eosinophils, Th2 cells, and group 2 innate lymphoid cells (ILC2s) [63,67]. Increased local concentrations of chemoattractants such as IL-3, IL-5, and eotaxin promote eosinophilic infiltration into the nasal mucosa [68]. The result of this infiltration is increased local concentrations of cytokines such as eotaxin, eosinophil cationic protein (ECP), IL-4, IL-5, IL-9, and IL-13 [63,67]. The symptoms in this phase include nasal blockage and watery nasal discharge [69].

Many different inhalant allergens are suspected to induce or exacerbate AR such as the pollen of various plants including Betulaceae, Oleaceae, Platanus, and Salicaceae; animal dander; fungal allergens; and air pollutants [70,71]. Recently, PM2.5 particles, along with other air pollutants, have been described as potential irritants that contribute to AR. In several studies, animal models of AR were thoroughly examined with regard to PM2.5 exposure and interesting results were obtained [26,72,73,74,75,76]. Increased sneezing, itching, and rhinorrhea are typical symptoms of AR after allergen presentation [65,69]. Several researchers have found that PM2.5 exposure also induced or exacerbated symptoms such as sneezing and nose rubbing in AR animal models [21,26,73,74,75].

A variety of data exists showing that PM2.5 exposure induces the activation of different cell types that are involved in AR pathogenesis. Wang et al. demonstrated that cytokines related to the epithelial cells such as TSLP and IL-33 were increased in the nasal mucosa of AR rats after PM2.5 exposure in comparison to the unexposed AR rats [26]. This observation may suggest an increased epithelial cell response which can lead to the intensified activation of DCs. Furthermore, nasal ECs became pro-inflammatory in AR mice exposed to PM2.5 in a study conducted by Piao et al. NF-kB was expressed mostly in the nucleus of nasal ECs and the highest count of NF-kB-stained cells was observed in mice with AR exacerbated by PM2.5. NF-kB plays an important role in the regulation of oxidative stress and inflammatory responses [75].

Th2 cells were also frequently reported to be more activated compared to Th1 cells after PM2.5 exposure in animal models of AR. Guo et al. investigated changes in Th1- and Th2-associated transcription factors after PM2.5 exposure. The results showed that AR rats exposed to PM2.5 had increased Th2-associated GATA-3 expression, whereas the expression of Th1-associated T-bet was decreased in comparison to the untreated AR rats [73]. Furthermore, Li et al. showed that PM2.5 exposure significantly decreased the percentage of Th1, but not Th2, cells in AR mice. Moreover, PM2.5 exposure altered the DNA methylation signatures of the IFN-γ promoter in the CD4+ T cells of AR mice, which might further decrease the percentage of Th1 cells. PM2.5 exposure also increased the activation of the ERK-DNMT pathway in CD4+ T cells, which suggests PM2.5 involvement in AR development [74]. Aside from gene expression, the proof for the intensified activation of Th2 cells was found in altered cytokine concentrations. In the sera of AR animals exposed to PM2.5, significant changes were found in comparison to the unexposed AR animals such as decreased levels of IFN-γ (Th1-related cytokine) and increased levels of IL-4, IL-5, IL-6, IL-13, and IL-25 (Th2 cytokines) [21,76]. Moreover, there is evidence for the increased infiltration of Th2 cells into the nasal mucosa of rats with AR exacerbated by PM2.5. Guo et al. showed increased levels of Th2-related cytokines such as IL-4 and IL-13 and decreased IFN-γ levels in the NLF of these animals [73]. In contrast, Wang et al. demonstrated an increased expression of IFN-γ in the nasal mucosa of rats with AR exacerbated by PM2.5. However, the researchers also observed an increased IL-4 level which was slightly higher than the IFN-γ level [26].

Recently, researchers have suggested that an imbalance between Th17 and Treg cells may also contribute to the pathogenesis of allergic disorders including AR [77]. Th17 cells can promote eosinophilic inflammation in AR whereas Treg cells may inhibit activation of Th2 cells, attenuating the response against the allergen [72,78]. In the NLF of AR animals exposed to PM2.5, increased levels of the Th17-related cytokine IL-17 and decreased levels of the Treg-related TGF-β1 and IL-10 were observed [75]. In contrast, Guo et al. demonstrated an increased TGF-β1 level in the NLF of rats with AR exacerbated by PM2.5 [73].

It was also shown that B cells involved in AR pathogenesis might be more numerous in the serum and intensively activated due to PM2.5 exposure. This suggestion was supported by elevated white blood cell counts in AR rats after PM2.5 exposure which was demonstrated by Wang et al. [26]. Moreover, serum IgE levels were higher in animals with AR exacerbated by PM2.5 which may suggest an increased activation of B cells [21,26,73].

Eosinophilic infiltration was also observed to be more severe in AR animals after PM2.5 exposure which was shown in NLF and histological samples [21,26,73,74,75,76]. Eotaxin, which is the main chemoattractant for eosinophils, was frequently reported to be increased in AR animals exposed to PM2.5. Increased serum levels of eotaxin-1 was reported by Wang et al. and Sun et al. [21,76]. Moreover, eotaxin was also elevated in NLF and nasal mucosa samples from AR animals exposed to PM2.5 [73,76].

The signs of increased infiltration were also observed by Wang et al. [26]. AR rats exposed to PM2.5 showed higher levels of ICAM-1 and VCAM-1, adhesion molecules involved in the first step of leukocyte infiltration, i.e., adhesion to the endothelium [26].

The process of autophagy was found to be increased in the nasal epithelia of patients suffering from AR [79]. Wang et al. demonstrated that autophagy was also increased in AR rats after PM2.5 exposure. The study showed that the expression of miR-338-3p in nasal epithelial cells was decreased after PM2.5 exposure. In vitro experiments revealed that miR-338-3p can regulate the AKT/mTOR pathway via interaction with UBE2Q1. The AKT/mTOR pathway is involved in the induction of autophagy processes. PM2.5 decreased miR-338-3p’s inhibitory effect on the AKT/mTOR pathway, resulting in autophagy induction in nasal epithelial cells which may play a role in AR development [80].

In addition, several studies have shown that PM2.5 induced mucosal changes such as vasodilatation, mucosal edema, and gland hyperplasia [21,26,76]. Moreover, the number of goblet cells was increased in the nasal mucosa of rats with AR exposed to PM2.5 [73]. Furthermore, Ouyang et al. showed that OVA-sensitized mice exposed to PM2.5 exhibited an increase in mucus production [81]. Increased mucus production plays a protective role; however, the overproduction may lead to a decrease in clearance and accelerate infections [45].

Interestingly, Yang et al. demonstrated that AR mice sensitized to house dust mites in combination with PM2.5 developed corticosteroid resistance that was not observed in AR mice sensitized with house dust mites alone. AR mice sensitized to house dust mites and PM2.5 had increased expression of Sos1 in their eosinophils. The study revealed that the higher expression of Sos1 led to a linkage between RAS and glucocorticoid receptor-α (GRα) in eosinophils that prevents bonding between steroids and GRα. Inactive GRα on eosinophils results in an absent or inadequate response to steroid therapy [82].

All these results suggest an important contribution of PM2.5 to AR pathogenesis. Interestingly, several epidemiological studies also confirmed the association of PM2.5 with AR. PM2.5 exposure was correlated with a higher incidence of AR in patients with Alzheimer’s disease [83]. Moreover, in Nanjing, China, PM2.5 exposure was positively correlated with the number of AR patients [30]. Finally, exposure to the higher concentration of PM2.5 in Hangzhou resulted in a higher risk of sensitization to house dust mites that may cause AR [84]. On the other hand, Dąbrowiecki et al. showed that the risk of AR hospitalizations in major Polish cities did not change significantly after PM2.5 exposure [85].

### 3.2. Rhinosinusitis

The nasal cavity is closely connected to the paranasal sinuses—structures responsible for warming and moistening inhaled air, proper air pressure equalization, smell, voice resonance as well as the protection sensitive skull structures. The surface of the sinuses is covered with the same airway epithelium as the nasal cavity and works as a buffer providing the first line of defense against environmental factors. Inhaled air pollution containing PM2.5 leads to multiple epithelial and sinus dysfunctions.

It is suggested that PM2.5 correlates with sinus disorders; however, it remains unknown whether air pollution leads to an exacerbation of previously existing illnesses or whether it contributes to disease development. The exact molecular mechanisms underlying the impact of PM2.5 on sinus functionality still require detailed investigations [86].

One of the most common diseases of the sinuses is rhinosinusitis in its acute or chronic form. Globally, chronic rhinosinusitis (CRS) affects almost 5–12% of the general population. The symptoms of CRS include nasal blockage, obstruction, congestion, nasal discharge, facial pain or pressure, and reduction in or loss of smell for more than 12 weeks [87]. CRS manifests as two main phenotypes: CRS with (CRSwNP) and without nasal polyps (CRSsNP). Recently, based on inflammatory response pathways, CRS has been divided into endotypes that are defined by the Th-related mechanisms and cytokine profiles. Additionally, the immune response pathways might be mixed and indicate geographical differences, suggesting a meaningful role of genetic, environmental, and dietary factors [86]. CRS in some cases also correlates with allergies, fungal inflammation, or eosinophilic infiltration.

The etiology of CRS is heterogenous and CRS is associated with chronic inflammation that has a poorly understood molecular background. Besides bacteria, viruses, fungi, and innate immune dysfunction, recent data point toward increasing air pollution as an important and highly pathogenic factor. Mady et al. observed that PM2.5 and black carbon (BC) correlated with CRS progression and exacerbation of clinically observed symptoms especially in patients with CRSsNP [22].

In the course of CRS, dysfunctions at the cellular level of the epithelium appear, similar to those observed in the in vitro studies describing the molecular effects of PM2.5 on airway epithelial cells: TJ and MCC dysfunction, microbiome dysbiosis, and goblet cell hyperplasia.

Studies have been shown that PM2.5 contributes to the secretion of pro-inflammatory factors and altered eosinophilic infiltration. Li et al. reported that PM leads to sinonasal inflammation, epithelial thickening, and the upregulation of eosinophils in the NLF [88]. In their retrospective study, Yang et al. were able to demonstrate a relationship between increased air pollution containing PM2.5 and eosinophilic CRSwNP as well as exacerbation of the disease [89]. Ramanathan et al. reported the destructive effect of PM2.5 on the sinus epithelium of mice and suggested its role in the development of nonallergic eosinophilic sinonasal inflammation. They observed an increased infiltration of neutrophils and macrophages, and eosinophilic inflammation with an upregulation of IL-13 and eotaxin-1 as well as other pro-inflammatory factors such as IL-1β and oncostatin M. The expression levels of proteins providing epithelial barrier integrity were downregulated including claudin-1, E-cadherin, IFN-γ, and IL-12p40 [24]. Similarly, Ma et al. demonstrated that the treatment of noninflamed nasal mucosa with PM2.5 and those obtained from eosinophilic CRS patients exhibited reduced TJ protein levels—claudin-1, zona occludens-1, and occludin. In the supernatant, IL-1α, IL-8, IL-10, and TIMP-1 (tissue inhibitor of metalloproteinase) were upregulated. Interestingly, these effects were partially attenuated with budesonide treatment [90]. In contrast, Patel et al. observed increased tissue inflammation and eosinophil infiltration levels in CRSwNP after ozone exposure and did not find histopathological changes associated with disease severity in the case of PM2.5 exposure [91].

Qing et al. observed an impact of PM2.5 on the mucosa of healthy individuals and patients with CRSwNP or AR. They report differences in the levels of pro-inflammatory cytokines, depending on the level of PM on that particular day. In the case of healthy individuals IL-6, IL-8, and TNF-α were upregulated in their nasal secretions on the days with high PM levels. In the CRSwNP and AR groups, increased levels of IL-6, IL-8, IL-1β, and IL-5 (a Th2-related cytokine) were observed [92].

Another effect of PM2.5 on the sinonasal epithelium is increased mucus production which might be involved in the MCC dysfunction. In a rabbit model of rhinosinusitis, Zhao et al. demonstrated that PM2.5 induces cilia morphological dysfunction or loss of cilia, goblet cell hyperplasia, collagen deposition, increased levels of fibroblasts and inflammatory cells, mucus overproduction, and tissue remodeling [93]. Additionally, Jiao et al. reported that even a short exposure to PM2.5 resulted in altered MUC5AC expression in nasal epithelial cells obtained from CRSwNP or control patients. MUC5AC is produced by goblet cells and is the main component of mucus and the authors demonstrated that this upregulation might be orchestrated by the EGFR-PI3K pathway [94]. Furthermore, it has been shown that PM2.5 might contribute to anosmia which is one of the widely observed clinical symptoms of CRS [95]. Elam et al. revealed that PM2.5 exposure contributed to CRS development in the active duty population. Interestingly, no correlation was found for PM10, ozone, or NO2 [29].

To alleviate the negative effects of PM2.5 on sinuses, it appears that it is crucial to discover the exact molecular mechanisms involved in epithelial damage and immune response pathways. Moreover, the seasonal changes in the composition of air pollution may alter the quality of these responses. The results of PM2.5 exposure in AR and Rhinosinusitis are summarized in Table 1.

## 4. Future Perspectives

This review emphasizes the importance of understanding the role of PM2.5 in the prevention and management of AR and rhinosinusitis. The current literature suggests that PM2.5 may contribute to the exacerbation of AR and may increase the risk of sensitization to common allergens such as house dust mites [30,83,84]. There is also evidence that PM2.5 may contribute to the pathogenesis of rhinosinusitis [86].

The literature emphasizes that there is an urgent need to create methods to alleviate the effects of PM2.5 exposure on the nasal epithelium to reduce the occurrence of AR and rhinosinusitis symptoms [84,86]. Recently, several compounds were tested for their potential to reduce the adverse effects of PM2.5. Sun et al. observed that AR rats gavaged with ursolic acid (UA) and exposed to PM2.5 exhibited decreased expression of Th2 cytokines, infiltration of eosinophils, and specific IgE production compared to a group of AR rats exposed to PM2.5 without UA treatment [76]. Moreover, PM2.5 induced remodeling of the nasal epithelium and increased mucus secretion in AR rats, which was also partially prevented by UA treatment [96]. Another compound tested to reduce the adverse effects of PM2.5 was rosmarinic acid (RosA). Zhou et al. demonstrated that the administration of RosA significantly decreased eosinophilic infiltration and reduced cell exfoliation in the nasal mucosa of AR rats. Moreover, the levels of OVA-specific IgE in the serum were significantly decreased after RosA treatment. The expression of IFN-γ was decreased and IL-4 and IL-13 levels were increased in AR rats exposed to PM2.5 while RosA administration reversed this trend. RosA also alleviated the change in expression of T-bet and GATA-3 mRNA and reversed the change in expression of NF-κB in the nasal mucosa after PM2.5 exposure [97].

Another potential strategy is to modulate the administration of approved drugs that target factors involved in PM2.5’s effects on CRS or AR. For instance, biological treatments that target the interleukins involved in PM2.5-modulated CRS could be given to patents with a higher PM2.5 exposure [98]. However, this strategy needs further extensive research on the molecular mechanisms behind PM2.5’s contribution to CRS or AR development and progression.

The other strategy to reduce the effects of PM2.5 on the nasal mucosa might be a restoration of the microbiome balance. Shen et al. investigated the effects of nasal treatment with PM2.5 on airway inflammation in healthy rats and the role of Broncho-Vaxom treatment after PM2.5 exposure. The authors were able to show that PM2.5 exposure triggered increased inflammatory cell infiltration, a higher number of Th17 cells, decreased levels of T regulatory cells (Tregs), and an upregulation of IL-4, IL-5, IL-13, and IL-17 and downregulation of IL-10. Interestingly, the administration of Broncho-Vaxom—a lysate of bacteria species—resulted in the downregulation of Th17 cells, IL-4, IL-5, IL-13, and IL-17 and an upregulation of IL-10 in comparison to the group exposed to PM2.5. Furthermore, they observed upregulated levels of aryl hydrocarbon receptor (AhR) in the PM2.5-treated rats; AhR is an immunoregulatory receptor involved in the response to environmental factors. These data suggest that the restoration of airway microbiome balance might be one of the pathways to overcome the negative effects of PM2.5 exposure [99].

The strategy to develop or use existing pharmacological compounds to alleviate the adverse effects of PM2.5 exposure is an interesting direction that, in the future, may become an important element of the treatment for patients with AR or rhinosinusitis who live in areas characterized by higher PM2.5 concentrations. More research is needed to develop and confirm the importance of this kind of potential medical intervention to fight the PM2.5-induced negative effects on the epithelia of the nasal and paranasal sinuses.

## 5. Conclusions

PM2.5 particles are certainly not only harmful to the lower respiratory tract, but also to the upper respiratory tract. High concentrations of PM2.5 potentially disrupt the organization of the nasal epithelium by damaging the intercellular connections, altering mitochondria metabolism, and increasing oxidative stress. Moreover, MCC, which is the main protective mechanism of the upper respiratory tract, may be altered due to PM2.5 exposure, ultimately leading to even more damage. People suffering from conditions such as AR and rhinosinusitis are particularly endangered by higher PM2.5 concentrations due to their potential contribution to the exacerbation of these conditions. There is also evidence that PM2.5 may contribute to the initial sensitization that may lead to AR development. In light of such evidence, there is a great need to develop new strategies against PM2.5 particles. More research is needed to better understand the mechanisms that are involved in the adverse effects of PM2.5 and to develop effective therapeutic strategies.

## Figures and Tables

**Table 1:** Comparison of PM2.5-induced changes in allergic rhinitis and rhinosinusitis.

Allergic Rhinitis		Rhinosinusitis	
PM2.5 effects on nasal and sinonasal mucosa			
Increased release of TSLP and IL-33 by nasal epithelial cells	[26]	Increased sinonasal inflammation and epithelial thickening	[88]
Increased NF-κB expression in nasal epithelial cells	[75]	Damage to sinonasal epithelium and cilia morphological dysfunction	[24,93]
Increased vasodilatation, mucosal edema, and gland hyperplasia	[21,26,76]	Downregulation of proteins involved in epithelial barrier integrity: - claudin-1 and E-cadherin - claudin-1, zona occludens-1, and occludin	[24,90]
Increased number of goblet cells and mucus production	[73,81]	Goblet cell hyperplasia and mucus overproduction Altered MUC5AC expression	[93,94]
Increased autophagy	[80]	Collagen deposition and increased levels of fibroblasts, tissue remodeling	[93]
**Infiltration of inflammatory cells into mucosa**			
↑ Adhesion molecules ICAM-1 and VCAM-1	[26]	Increased migration factors: IL-8	[90]
Increased infiltration of eosinophils	[21,26,73,74,75,76]	Increased infiltration of neutrophils and macrophages	[24]
**PM2.5 effect on immunological processes**			
Decreased Th1 cell activity: - ↓ T-bet expression - ↓ Th1 cells percentage - ↓ IFN-γ	[21,73]	Decreased IFN-γ and IL-12p40	[24]
Increased Th2 cell activity: - ↑ GATA3 expression - ↑ percentage of Th2 cells - ↑ IL-4, IL-5, IL-6, IL-13, IL-25	[21,73,76]	Increased IL-5, IL-6	[92]
Increased Th17 cell activity	[75]		
Decreased Treg cell activity: - ↓ IL-10	[75]	Increased IL-10	[90]
Increased B cell activity: - Higher B cell count - ↑ IgE	[21,26,73]		
Increased activity of eosinophils: - ↑ Eotaxin	[21,73,76]	Increased activity of eosinophils: ↑ IL-13, eotaxin-1	[88]
		Increased pro-inflammatory factors: - IL-1β and oncostatin M - IL-1α, IL-6 - TIMP1	[24,90,92]

## Data Availability

Available on request from the corresponding authors.

## Abbreviations

AhR	Aryl hydrocarbon receptor
AJs	Adherence junctions
AKT	Protein kinase B
AMPKα	AMP-activated protein kinase alpha
AR	Allergic rhinitis
ARIA	Allergic Rhinitis and its impact on Asthma classification
BC	Black carbon
CAT	Catalase
CBF	Ciliary beat frequency
CCL20	Chemokine (C-C motif) ligand 20
CLDN	Claudin
CLDN-1	Claudin-1
CLDN-4	Claudin-4
CLDN-7	Claudin-7
CO	Carbon monoxide
COPD	Chronic obstructive pulmonary disease
COVID-19	Coronavirus disease 2019
CRS	Chronic rhinosinusitis
CRSsNP	Chronic rhinosinusitis without nasal polyps
CRSwNP	Chronic rhinosinusitis with nasal polyps
DCs	Dendritic cells
DNA	Deoxyribonucleic acid
DNMT	DNA methyltransferase
Drp1	Dynamin-related protein 1
ECP	Eosinophil cationic protein
ECs	Epithelial cells
EGFR	Epidermal growth factor receptor
ERK	Extracellular signal-activated kinase
Fis1	Mitochondrial fission 1 protein
FTH1	Ferritin heavy chain 1
FTL	Ferritin light chain
GATA-3	GATA binding protein 3
GM-CSF	Granulocyte–macrophage colony-stimulating factor
GPx4	Glutathione peroxidase 4
GRα	Glucocorticoid receptor-alpha
GSH-Px	Glutathione peroxidase
H&E	Hematoxylin and eosin
HNEpCs	Human nasal epithelial cells
ICAM-1	Intercellular adhesion molecule 1
IgE	Immunoglobulin E
IL-1α	Interleukin-1 alpha
IL-1 β	Interleukin-1 beta
IL-4	Interleukin 4
IL-5	Interleukin 5
IL-6	Interleukin 6
IL-8	Interleukin 8
IL-10	Interleukin 10
IL-12p40	The p40 subunit of interleukin 12
IL-13	Interleukin 13
IL-17	Interleukin 17
IL-25	Interleukin 25
IL-33	Interleukin 33
ILC2s	Group 2 innate lymphoid cells
IFN-γ	Interferon gamma
MCC	Mucociliary clearance
MDA	Malondialdehyde
MFN1	Mitofusin 1
miR	microRNA
mRNAs	Messenger ribonucleic acid
mTOR	Mammalian target of rapamycin
MUC5A	Mucin 5A
MUC5B	Mucin 5B
MUC5AC	Mucin 5AC
NaCl	Sodium chloride
NF-kB	Nuclear factor kappa-light-chain-enhancer of activated B cells
NLF	Nasal lavage fluid
NO2	Nitrogen dioxide
Nrf2	Nuclear factor erythroid 2-related factor 2
OPA1	Mitochondrial dynamin-like GTPase
OVA	Ovalbumin
PI3K	Phosphoinositide 3-kinase
PM	Particulate matter
ROS	Reactive oxygen species
RosA	Rosmarinic acid
SO2	Sulfur dioxide
SOD	Superoxide dismutase
Sos1	Son of sevenless 1
T-bet	T-box transcription factor TBX21
TGF-β1	Transforming growth factor beta 1
Th1	T helper 1 cells
Th2	T helper 2 cells
Th17	T helper 17 cells
Treg	T regulatory cells
TIMP-1	Tissue inhibitor of metalloproteinase
TJs	Tight junctions
TNF-α	Tumor necrosis factor alpha
TSLP	Thymic stromal lymphopoietin
UA	Ursolic Acid
UBE2Q1	A member of ubiquitin-conjugating enzyme family
VCAM-1	Vascular cell adhesion molecule 1
WHO	World Health Organization
xCT	SLC7A11—cystine/glutamate antiporter
ZO	Zonula occludens
ZO-1	Zonula occludens 1
ZO-2	Zonula occludens 2

## References

[1] Air Pollution. https://www.who.int/health-topics/air-pollution#tab=tab_1.

[2] Katsouyanni K. (2003). Ambient air pollution and health. Br. Med. Bull..

[3] Mannucci P.M., Franchini M. (2017). Health Effects of Ambient Air Pollution in Developing Countries. Int. J. Environ. Res. Public Health.

[4] Losacco C., Perillo A. (2018). Particulate matter air pollution and respiratory impact on humans and animals. Environ. Sci. Pollut. Res. Int..

[5] Saraga D., Maggos T., Degrendele C., Klanova J., Horvat M., Kocman D., Kanduc T., Garcia Dos Santos S., Franco R., Gomez P.M. (2021). Multi-city comparative PM_2.5_ source apportionment for fifteen sites in Europe: The ICARUS project. Sci. Total Environ..

[6] Salameh D., Detournay A., Pey J., Pérez N., Liguori F., Saraga D., Bove M.C., Brotto P., Cassola F., Massabò D. (2015). PM_2.5_ chemical composition in five European Mediterranean cities: A 1-year study. Atmos. Res..

[7] Majumdar D. (2021). How are the Two Most Polluted Metro-cities of India Combating Air Pollution? Way Forward after Lifting of COVID-19 Lockdown. Aerosol Air Qual. Res..

[8] Xu L., Chen X., Chen J., Zhang F., He C., Zhao J., Yin L. (2012). Seasonal variations and chemical compositions of PM_2.5_ aerosol in the urban area of Fuzhou, China. Atmos. Res..

[9] Juda-Rezler K., Zajusz-Zubek E., Reizer M., Maciejewska K., Kurek E., Bulska E., Klejnowski K. (2021). Bioavailability of elements in atmospheric PM_2.5_ during winter episodes at Central Eastern European urban background site. Atmos. Environ..

[10] Chauhan A., Singh R.P. (2020). Decline in PM_2.5_ concentrations over major cities around the world associated with COVID-19. Environ. Res..

[11] Xing Y.F., Xu Y.H., Shi M.H., Lian Y.X. (2016). The impact of PM_2.5_ on the human respiratory system. J. Thorac. Dis..

[12] Wang C., Tu Y., Yu Z., Lu R. (2015). PM_2.5_ and Cardiovascular Diseases in the Elderly: An Overview. Int. J. Environ. Res. Public. Health.

[13] Carre J., Gatimel N., Moreau J., Parinaud J., Leandri R. (2017). Does air pollution play a role in infertility?: A systematic review. Environ. Health.

[14] Yang L., Li C., Tang X. (2020). The Impact of PM_2.5_ on the Host Defense of Respiratory System. Front. Cell Dev. Biol..

[15] Liang S., Zhang J., Ning R., Du Z., Liu J., Batibawa J.W., Duan J., Sun Z. (2020). The critical role of endothelial function in fine particulate matter-induced atherosclerosis. Part. Fibre Toxicol..

[16] Zhang S., Routledge M.N. (2020). The contribution of PM_2.5_ to cardiovascular disease in China. Environ. Sci. Pollut. Res. Int..

[17] Orellano P., Quaranta N., Reynoso J., Balbi B., Vasquez J. (2017). Effect of outdoor air pollution on asthma exacerbations in children and adults: Systematic review and multilevel meta-analysis. PLoS ONE.

[18] Ni L., Chuang C.C., Zuo L. (2015). Fine particulate matter in acute exacerbation of COPD. Front. Physiol..

[19] Li R., Zhou R., Zhang J. (2018). Function of PM_2.5_ in the pathogenesis of lung cancer and chronic airway inflammatory diseases. Oncol. Lett..

[20] Hamra G.B., Guha N., Cohen A., Laden F., Raaschou-Nielsen O., Samet J.M., Vineis P., Forastiere F., Saldiva P., Yorifuji T. (2014). Outdoor particulate matter exposure and lung cancer: A systematic review and meta-analysis. Environ. Health Perspect..

[21] Wang J., Guo Z., Zhang R., Han Z., Huang Y., Deng C., Dong W., Zhuang G. (2020). Effects of N-acetylcysteine on oxidative stress and inflammation reactions in a rat model of allergic rhinitis after PM_2.5_ exposure. Biochem. Biophys. Res. Commun..

[22] Mady L.J., Schwarzbach H.L., Moore J.A., Boudreau R.M., Tripathy S., Kinnee E., Dodson Z.M., Willson T.J., Clougherty J.E., Lee S.E. (2018). Air pollutants may be environmental risk factors in chronic rhinosinusitis disease progression. Int. Forum Allergy Rhinol..

[23] Xian M., Ma S., Wang K., Lou H., Wang Y., Zhang L., Wang C., Akdis C.A. (2020). Particulate Matter 2.5 Causes Deficiency in Barrier Integrity in Human Nasal Epithelial Cells. Allergy Asthma Immunol. Res..

[24] Ramanathan M., London N.R., Tharakan A., Surya N., Sussan T.E., Rao X., Lin S.Y., Toskala E., Rajagopalan S., Biswal S. (2017). Airborne Particulate Matter Induces Nonallergic Eosinophilic Sinonasal Inflammation in Mice. Am. J. Respir. Cell Mol. Biol..

[25] Guo Z., Hong Z., Dong W., Deng C., Zhao R., Xu J., Zhuang G., Zhang R. (2017). PM_2.5_-Induced Oxidative Stress and Mitochondrial Damage in the Nasal Mucosa of Rats. Int. J. Environ. Res. Public Health.

[26] Wang Y.-L., Gao W., Li Y., Wang Y.-F. (2017). Concentration-dependent effects of PM_2.5_ mass on expressions of adhesion molecules and inflammatory cytokines in nasal mucosa of rats with allergic rhinitis. Eur. Arch. Oto-Rhino-Laryngol..

[27] Mariani J., Favero C., Spinazze A., Cavallo D.M., Carugno M., Motta V., Bonzini M., Cattaneo A., Pesatori A.C., Bollati V. (2018). Short-term particulate matter exposure influences nasal microbiota in a population of healthy subjects. Environ. Res..

[28] Qin T., Zhang F., Zhou H., Ren H., Du Y., Liang S., Wang F., Cheng L., Xie X., Jin A. (2019). High-Level PM_2.5_/PM_10_ Exposure Is Associated With Alterations in the Human Pharyngeal Microbiota Composition. Front. Microbiol..

[29] Elam T., Raiculescu S., Biswal S., Zhang Z., Orestes M., Ramanathan M. (2022). Air Pollution Exposure and the Development of Chronic Rhinosinusitis in the Active Duty Population. Mil. Med..

[30] Chu H., Xin J., Yuan Q., Wang M., Cheng L., Zhang Z., Lu M. (2019). The effects of particulate matters on allergic rhinitis in Nanjing, China. Environ. Sci. Pollut. Res. Int..

[31] Duchen M.R. (2004). Roles of mitochondria in health and disease. Diabetes.

[32] Pole A., Dimri M., Dimri G.P. (2016). Oxidative stress, cellular senescence and ageing. AIMS Mol. Sci..

[33] Kim K.A., Jung J.H., Kang I.G., Choi Y.S., Kim S.T. (2018). ROS Is Involved in Disruption of Tight Junctions of Human Nasal Epithelial Cells Induced by HRV16. Laryngoscope.

[34] Yoon Y.H., Yeon S.H., Choi M.R., Jang Y.S., Kim J.A., Oh H.W., Jun X., Park S.K., Heo J.Y., Rha K.S. (2020). Altered Mitochondrial Functions and Morphologies in Epithelial Cells Are Associated With Pathogenesis of Chronic Rhinosinusitis With Nasal Polyps. Allergy Asthma Immunol. Res..

[35] Hong Z., Guo Z., Zhang R., Xu J., Dong W., Zhuang G., Deng C. (2016). Airborne Fine Particulate Matter Induces Oxidative Stress and Inflammation in Human Nasal Epithelial Cells. Tohoku J. Exp. Med..

[36] Hong Z., Zeng P., Zhuang G., Guo Q., Cai C. (2021). Toxicological Effects of Artificial Fine Particulate Matter in Rats through Induction of Oxidative Stress and Inflammation. Tohoku J. Exp. Med..

[37] Ma Q. (2013). Role of nrf2 in oxidative stress and toxicity. Annu. Rev. Pharmacol. Toxicol..

[38] Gu W., Hou T., Zhou H., Zhu L., Zhu W., Wang Y. (2023). Ferroptosis is involved in PM_2.5_-induced acute nasal epithelial injury via AMPK-mediated autophagy. Int. Immunopharmacol..

[39] Fariss M.W., Chan C.B., Patel M., Van Houten B., Orrenius S. (2005). Role of mitochondria in toxic oxidative stress. Mol. Interv..

[40] Jia J., Xia J., Zhang R., Bai Y., Liu S., Dan M., Li T., Yan T., Chen L., Gong S. (2019). Investigation of the impact of PM_2.5_ on the ciliary motion of human nasal epithelial cells. Chemosphere.

[41] Zhao R., Guo Z., Zhang R., Deng C., Xu J., Dong W., Hong Z., Yu H., Situ H., Liu C. (2018). Nasal epithelial barrier disruption by particulate matter ≤2.5 μm via tight junction protein degradation. J. Appl. Toxicol..

[42] Reddy P.H. (2011). Mitochondrial Dysfunction and Oxidative Stress in Asthma: Implications for Mitochondria-Targeted Antioxidant Therapeutics. Pharmaceuticals.

[43] Jiao J., Wang C., Zhang L. (2019). Epithelial physical barrier defects in chronic rhinosinusitis. Expert. Rev. Clin. Immunol..

[44] Kojima T., Go M., Takano K., Kurose M., Ohkuni T., Koizumi J., Kamekura R., Ogasawara N., Masaki T., Fuchimoto J. (2013). Regulation of tight junctions in upper airway epithelium. Biomed. Res. Int..

[45] Zhang N., Van Crombruggen K., Gevaert E., Bachert C. (2016). Barrier function of the nasal mucosa in health and type-2 biased airway diseases. Allergy.

[46] Jang A.S. (2014). The apical junctional complex in respiratory diseases. Chonnam Med. J..

[47] Yasuda M., Inui T.A., Hirano S., Asano S., Okazaki T., Inui T., Marunaka Y., Nakahari T. (2020). Intracellular Cl^−^ Regulation of Ciliary Beating in Ciliated Human Nasal Epithelial Cells: Frequency and Distance of Ciliary Beating Observed by High-Speed Video Microscopy. Int. J. Mol. Sci..

[48] Bergougnoux A., Claustres M., De Sario A. (2015). Nasal epithelial cells: A tool to study DNA methylation in airway diseases. Epigenomics.

[49] Rubin B.K. (2002). Physiology of airway mucus clearance. Respir. Care.

[50] Jiao J., Zhang L. (2019). Influence of Intranasal Drugs on Human Nasal Mucociliary Clearance and Ciliary Beat Frequency. Allergy Asthma Immunol. Res..

[51] Sobiesk J.L., Munakomi S. (2022). Anatomy, Head and Neck, Nasal Cavity.

[52] Kaliner M., Marom Z., Patow C., Shelhamer J. (1984). Human respiratory mucus. J. Allergy Clin. Immunol..

[53] Groneberg D.A., Peiser C., Dinh Q.T., Matthias J., Eynott P.R., Heppt W., Carlstedt I., Witt C., Fischer A., Chung K.F. (2003). Distribution of respiratory mucin proteins in human nasal mucosa. Laryngoscope.

[54] Camargo Pires-Neto R., Júlia Lichtenfels A., Regina Soares S., Macchione M., Hilário Nascimento Saldiva P., Dolhnikoff M. (2006). Effects of São Paulo air pollution on the upper airways of mice. Environ. Res..

[55] Shah V.S., Meyerholz D.K., Tang X.X., Reznikov L., Abou Alaiwa M., Ernst S.E., Karp P.H., Wohlford-Lenane C.L., Heilmann K.P., Leidinger M.R. (2016). Airway acidification initiates host defense abnormalities in cystic fibrosis mice. Science.

[56] Paplinska-Goryca M., Misiukiewicz-Stepien P., Proboszcz M., Nejman-Gryz P., Gorska K., Zajusz-Zubek E., Krenke R. (2021). Interactions of nasal epithelium with macrophages and dendritic cells variously alter urban PM-induced inflammation in healthy, asthma and COPD. Sci. Rep..

[57] Janssen N.A.H., Strak M., Yang A., Hellack B., Kelly F.J., Kuhlbusch T.A.J., Harrison R.M., Brunekreef B., Cassee F.R., Steenhof M. (2015). Associations between three specific a-cellular measures of the oxidative potential of particulate matter and markers of acute airway and nasal inflammation in healthy volunteers. Occup. Environ. Med..

[58] Hatakeyama N., Matsuda N. (2014). Alert cell strategy: Mechanisms of inflammatory response and organ protection. Curr. Pharm. Des..

[59] Hoyte F.C.L., Nelson H.S. (2018). Recent advances in allergic rhinitis. F1000Research.

[60] Meltzer E.O. (2016). Allergic Rhinitis: Burden of Illness, Quality of Life, Comorbidities, and Control. Immunol. Allergy Clin. N. Am..

[61] Kakli H.A., Riley T.D. (2016). Allergic Rhinitis. Prim. Care.

[62] Bousquet J., Anto J.M., Bachert C., Baiardini I., Bosnic-Anticevich S., Walter Canonica G., Melen E., Palomares O., Scadding G.K., Togias A. (2020). Allergic rhinitis. Nat. Rev. Dis. Primers.

[63] Drazdauskaite G., Layhadi J.A., Shamji M.H. (2020). Mechanisms of Allergen Immunotherapy in Allergic Rhinitis. Curr. Allergy Asthma Rep..

[64] Bernstein D.I., Schwartz G., Bernstein J.A. (2016). Allergic Rhinitis: Mechanisms and Treatment. Immunol. Allergy Clin. N. Am..

[65] Small P., Keith P.K., Kim H. (2018). Allergic rhinitis. Allergy Asthma Clin. Immunol..

[66] Watts A.M., Cripps A.W., West N.P., Cox A.J. (2019). Modulation of Allergic Inflammation in the Nasal Mucosa of Allergic Rhinitis Sufferers With Topical Pharmaceutical Agents. Front. Pharmacol..

[67] Scadding G.W., Eifan A., Penagos M., Dumitru A., Switzer A., McMahon O., Phippard D., Togias A., Durham S.R., Shamji M.H. (2015). Local and systemic effects of cat allergen nasal provocation. Clin. Exp. Allergy.

[68] Baraniuk J.N. (1997). Pathogenesis of allergic rhinitis. J. Allergy Clin. Immunol..

[69] Eifan A.O., Durham S.R. (2016). Pathogenesis of rhinitis. Clin. Exp. Allergy.

[70] Costache A., Berghi O.N., Cergan R., Dumitru M., Neagos A., Popa L.G., Giurcaneanu C., Vrinceanu D. (2021). Respiratory allergies: Salicaceae sensitization (Review). Exp. Ther. Med..

[71] Wise S.K., Lin S.Y., Toskala E., Orlandi R.R., Akdis C.A., Alt J.A., Azar A., Baroody F.M., Bachert C., Canonica G.W. (2018). International Consensus Statement on Allergy and Rhinology: Allergic Rhinitis. Int. Forum Allergy Rhinol..

[72] Wang Y.X., Gu Z.W., Cao Z.W. (2021). Difference between CD25^+^Tregs and Helios^+^Tregs in a murine model of allergic rhinitis. Braz. J. Otorhinolaryngol..

[73] Guo Z.-Q., Dong W.-Y., Xu J., Hong Z.-C., Zhao R.-W., Deng C.-R., Zhuang G.-S., Zhang R.-X. (2017). T-helper type 1-T-helper type 2 shift and nasal remodeling after fine particulate matter exposure in a rat model of allergic rhinitis. Am. J. Rhinol. Allergy.

[74] Li Y., Zhou J., Rui X., Zhou L., Mo X. (2019). PM_2.5_ exposure exacerbates allergic rhinitis in mice by increasing DNA methylation in the IFN-γ gene promoter in CD4+T cells via the ERK-DNMT pathway. Toxicol. Lett..

[75] Piao C.H., Fan Y., Nguyen T.V., Shin H.S., Kim H.T., Song C.H., Chai O.H. (2021). PM_2.5_ Exacerbates Oxidative Stress and Inflammatory Response through the Nrf2/NF-κB Signaling Pathway in OVA-Induced Allergic Rhinitis Mouse Model. Int. J. Mol. Sci..

[76] Sun N., Han Z., Wang H., Guo Z., Deng C., Dong W., Zhuang G., Zhang R. (2020). Effects of Ursolic Acid on the Expression of Th1-Th2-related Cytokines in a Rat Model of Allergic Rhinitis After PM_2.5_ Exposure. Am. J. Rhinol. Allergy.

[77] Wang J., Qiu L., Chen Y., Chen M. (2021). Sublingual immunotherapy increases Treg/Th17 ratio in allergic rhinitis. Open Med. (Wars).

[78] Liu Y., Zeng M., Liu Z. (2015). Th17 response and its regulation in inflammatory upper airway diseases. Clin. Exp. Allergy.

[79] Li J., Li Y. (2019). Autophagy is involved in allergic rhinitis by inducing airway remodeling. Int. Forum Allergy Rhinol..

[80] Wang J.C., Huang Y., Zhang R.X., Han Z.J., Zhou L.L., Sun N., Dong W.Y., Zhuang G.S. (2021). miR-338-3p inhibits autophagy in a rat model of allergic rhinitis after PM_2.5_ exposure through AKT/mTOR signaling by targeting UBE2Q1. Biochem. Biophys. Res. Commun..

[81] Ouyang Y., Xu Z., Fan E., Li Y., Miyake K., Xu X., Zhang L. (2018). Changes in gene expression in chronic allergy mouse model exposed to natural environmental PM_2.5_-rich ambient air pollution. Sci. Rep..

[82] Yang G., Suo L.M., Geng X.R., Zeng X.H., Liu J.Q., Liu Z.Q., Li M., Chen Y.R., Hong J.Y., Xue J.M. (2022). An eosinophil-Sos1-RAS axis licenses corticosteroid resistance in patients with allergic rhinitis. Immunobiology.

[83] Li R.L., Ho Y.C., Luo C.W., Lee S.S., Kuan Y.H. (2019). Influence of PM_2.5_ Exposure Level on the Association between Alzheimer’s Disease and Allergic Rhinitis: A National Population-Based Cohort Study. Int. J. Environ. Res. Public Health.

[84] Ye Q., Zhang T., Mao J.H. (2020). Haze facilitates sensitization to house dust mites in children. Environ. Geochem. Health.

[85] Dabrowiecki P., Chcialowski A., Dabrowiecka A., Piorkowska A., Badyda A. (2023). Exposure to ambient air pollutants and short-term risk for exacerbations of allergic rhinitis: A time-stratified, case-crossover study in the three largest urban agglomerations in Poland. Respir. Physiol. Neurobiol..

[86] Leland E.M., Zhang Z., Kelly K.M., Ramanathan M. (2021). Role of Environmental Air Pollution in Chronic Rhinosinusitis. Curr. Allergy Asthma Rep..

[87] Fokkens W.J., Lund V.J., Hopkins C., Hellings P.W., Kern R., Reitsma S., Toppila-Salmi S., Bernal-Sprekelsen M., Mullol J., Alobid I. (2020). European Position Paper on Rhinosinusitis and Nasal Polyps 2020. Rhinology.

[88] Li N., Wang M., Bramble L.A., Schmitz D.A., Schauer J.J., Sioutas C., Harkema J.R., Nel A.E. (2009). The adjuvant effect of ambient particulate matter is closely reflected by the particulate oxidant potential. Environ. Health Perspect..

[89] Yang X., Shen S., Deng Y., Wang C., Zhang L. (2021). Air Pollution Exposure Affects Severity and Cellular Endotype of Chronic Rhinosinusitis with Nasal Polyps. Laryngoscope.

[90] Ma S., Xian M., Wang Y., Wang C., Zhang L. (2021). Budesonide repairs decreased barrier integrity of eosinophilic nasal polyp epithelial cells caused by PM_2.5_. Clin. Transl. Allergy.

[91] Patel T.R., Tajudeen B.A., Brown H., Gattuso P., LoSavio P., Papagiannopoulos P., Batra P.S., Mahdavinia M. (2021). Association of Air Pollutant Exposure and Sinonasal Histopathology Findings in Chronic Rhinosinusitis. Am. J. Rhinol. Allergy.

[92] Qing H., Wang X., Zhang N., Zheng K., Du K., Zheng M., Li Y., Chang Y., Zhang L., Bachert C. (2019). The Effect of Fine Particulate Matter on the Inflammatory Responses in Human Upper Airway Mucosa. Am. J. Respir. Crit. Care Med..

[93] Zhao R., Guo Z., Dong W., Deng C., Han Z., Liu J., Wang H., Zhuang G., Zhang R. (2018). Effects of PM_2.5_ on mucus secretion and tissue remodeling in a rabbit model of chronic rhinosinusitis. Int. Forum Allergy Rhinol..

[94] Jiao J., Hu P., Li Y., Cai C., Wang X., Zhang L. (2021). PM_2.5_ Upregulates the Expression of MUC5AC via the EGFR-PI3K Pathway in Human Sinonasal Epithelial Cells. Int. Arch. Allergy Immunol..

[95] Zhang Z., Rowan N.R., Pinto J.M., London N.R., Lane A.P., Biswal S., Ramanathan M. (2021). Exposure to Particulate Matter Air Pollution and Anosmia. JAMA Netw. Open.

[96] Sun N., Deng C., Zhao Q., Han Z., Guo Z., Wang H., Dong W., Duan Y., Zhuang G., Zhang R. (2021). Ursolic Acid Alleviates Mucus Secretion and Tissue Remodeling in Rat Model of Allergic Rhinitis After PM_2.5_ Exposure. Am. J. Rhinol. Allergy.

[97] Zhou L., Huang Y., Han Z., Wang J., Sun N., Zhang R., Dong W., Deng C., Zhuang G. (2022). Effects of rosmarinic acid on the inflammatory response in allergic rhinitis rat models after PM_2.5_ exposure. J. Clin. Lab. Anal..

[98] Cergan R., Berghi O.N., Dumitru M., Vrinceanu D., Manole F., Serboiu C.S. (2023). Biologics for Chronic Rhinosinusitis-A Modern Option for Therapy. Life.

[99] Shen Y., Zhang Z.-H., Hu D., Ke X., Gu Z., Zou Q.-Y., Hu G.-H., Song S.-H., Kang H.-Y., Hong S.-L. (2018). The airway inflammation induced by nasal inoculation of PM_2.5_ and the treatment of bacterial lysates in rats. Sci. Rep..

